# The *values* and *principles* underpinning community engagement approaches to tackling antimicrobial resistance (AMR)

**DOI:** 10.1080/16549716.2020.1837484

**Published:** 2020-11-17

**Authors:** Jessica Mitchell, Paul Cooke, Sushil Baral, Naomi Bull, Catherine Stones, Emmanuel Tsekleves, Nervo Verdezoto, Abriti Arjyal, Romi Giri, Ashim Shrestha, Rebecca King

**Affiliations:** a Centre for World Cinemas and Digital Cultures, Faculty of Arts, Humanities and Cultures, University of Leeds, Woodhouse, UK; b University of Leeds, Woodhouse, UK; c Nuffield Centre for International Health and Development, Worsley Building University of Leeds, Woodhouse, England; d HERD International, Kathmandu, Nepal; e London School of Hygiene and Tropical Medicine, London, UK; f School of Design, University of Leeds, Woodhouse, UK; g ImaginationLancaster, LICA, Lancaster University, Lancaster, UK; h School of Computer Science and Informatics, Cardiff University, Cardiff, UK

**Keywords:** Antimicrobial Resistance, One health, participation, equity, tool, antibiotic

## Abstract

This paper presents seven *values* underpinning the application of Community Engagement (CE) approaches to the One Health challenge of antimicrobial resistance (AMR) developed during an international workshop in June 2019. We define a *value* as a quality or standard which a CE project is aiming for, whilst a *principle* is an objective which underpins the value and facilitates its achievement. The *values* of Clarity, Creativity, (being) Evidence-led, Equity, Interdisciplinarity, Sustainability and Flexibility were identified by a network of 40 researchers and practitioners who utilise CE approaches to tackle complex One Health challenges including, but not limited to, AMR. We present our understanding of these seven *values* and their underlying *principles* as a flexible tool designed to support stakeholders within CE for AMR projects. We include practical guidance on working toward each *value*, plus case studies of the *values* in action within existing AMR interventions. Finally, we consider the extent to which CE approaches are appropriate to tackle AMR challenges. We reflect on these in relation to the tool, and current literature for both CE and AMR research. Authors and co-producers anticipate this tool being used to scene-set, road map and trouble shoot the development, implementation, and evaluation of CE projects to address AMR and other One Health challenges. However, the tool is not prescriptive but responsive to the context and needs of the community, opening opportunity to build a truly collaborative and community-centred approach to AMR research.

## Background

### Introduction to our context and co-producers

Antimicrobial resistance (AMR) is the process by which microbes (including bacteria, viruses, fungi, parasites) change or evolve to survive the drugs used to destroy them. Although naturally occurring, AMR is accelerating on a global scale due to the overuse, misuse, and inappropriate disposal of antimicrobials. It is considered a One Health issue because it impacts humans, animals and the environment and requires cross-sector collaboration to tackle [1]. Without action this decade, AMR could cause economic damage on a similar scale to the 2008 financial crisis, leading to 300 million deaths by 2050 [2] and pushing 28.3 million people into poverty, the bulk of which (26.2 million) will inevitably reside in low-middle income countries (LMICs) [3]. Such countries face major inequalities in health care, wide economic disparity, governmental corruption leading to poor return on taxation, and poor hygiene and sanitation systems [4,5], meaning they also stand to experience the highest death rates attributed to AMR. As AMR is a major threat to global health, food production and economic stability, many researcher teams seek to address it via the production of new drugs and top-down policy changes on antimicrobial use [6,7]. However, AMR is also a social issue driven by human behaviour, and thus others are attempting to tackle it via engaging with communities. Such bottom-up approaches can explore the local context of antimicrobial use which, in turn, can facilitate the co-development of bespoke solutions to minimise AMR in that community. The benefits of such community engagement (CE) appear particularly meaningful when considering LMICs, as the local specificity of this approach can take into account many of the complex AMR-related inequalities detailed above [8].

There is a growing literature that discusses the potential of CE in tackling Health challenges in LMICs [9–12]. However, to the best of our knowledge there is no current guidance on applying CE methods, specifically, to AMR. This may be because AMR has historically been viewed as a biological problem requiring top-down solutions including policy and system-level changes [6]. Utilising a bottom-up approach such as CE can be challenging considering the dynamic nature of AMR. Firstly, for example, in many projects the local drivers of resistance may not be fully understood by the research team, and information given to the community may change during the project. This has the potential to create mistrust between community and researchers and it can also conflict with existing CE frameworks. Considering The Ladder of Participation by Arnstein [13] in the scope of AMR, the community may not ever be in full, or even delegated, control of the process because of the changing local AMR information they receive . There will also be periods within a CE for AMR intervention that information must be given to the community in a one-way process, whilst misinformation at community level must be corrected by the research team. This practice is essential if AMR is to be tackled in a given community, but can potentially inhibit the community learning for itself, a problem recognised across health-based applications of CE [14]. Secondly, AMR remains a One Health problem. when attempting to tackle AMR research, teams must consider behaviours beyond human health for example; agricultural, veterinary and environmental practices. Frameworks to support such interdisciplinary reach are lacking. Finally, there is limited consensus on whether CE can be successful in addressing AMR. Evaluations tend to focus on changes in knowledge, attitudes, and practice and several studies suggest that simply raising awareness of AMR alone is not enough to create a change in practice or behaviour [11,15,16]. Measuring behaviour change itself is challenging and often based on self-reported data which raises questions around validity for some academic disciplines and policy makers [17]. Considering these complexities, researchers applying CE to AMR and One Health challenges require additional guidance to ensure their interventions are as informative, engaging and well evidenced as possible.

This article discusses the creation of a flexible tool to guide the application of CE methods to AMR or broader One Health research. The tool was compiled based on workshop discussions by a range of researchers, practitioners and government officials. It draws on the collective experience of these co-producers, summarising this into seven *values* which should be considered when developing, implementing, and evaluating CE interventions within One Health. The tool also presents key *principles*, which act as indicators as to whether each *value* is being realised. By utilising similar language to existing frameworks this tool could be implemented alongside them. However, it also provides a holistic One Health view of the CE method through discussion of specific *values* and *principles* implicated in AMR research. We anticipate users of the tool to be those working on either AMR or another complex One Health challenge who wish to utilise methods which truly engage the focal community they are aiming to support.

### Our co-producers

In June 2019 a group of 40 researchers and practitioners met in Kathmandu, Nepal for a three-day workshop designed to discuss the role of community engagement in tackling AMR. The group was convened by a partnership between HERD International and the University of Leeds, and aimed to bring together teams utilising CE methods in AMR and wider health research. Invited delegates represented 20 projects delivered by 18 organisations, including Universities, NGOs, and local government, with interventions spread across 10 LMICs. From here on we refer to these delegates as our co-producers. While some were experts in various dimensions of AMR, others brought insights from other One Health issues. Disciplinary representation was broad and included those in the field of medicine, social science, the arts, and animal health. What bound co-producers were their interests and expertise in community-level research across One Health challenges which ranged from the use of participatory theatre to improve mental health, to discussing AMR with children via grassroots comics. Their diversity provided rich discussions around what constitutes successful CE and how it can be applied to AMR. This paper synthesises discussions into a practical tool detailing seven key *values* and their underpinning *principles* which should facilitate and support the successful application of CE to AMR. We also exemplify these *values* in action through four Case Studies.

### Our definition of community engagement

A major step in the process of creating the tool was to clarify our co-producers’ shared definition of CE. From our perspective CE is a specific type of intervention within the wider community-based participatory research (CBPR) continuum [18,19]. It involves the research team immersing themselves within the community to better understand, and eventually tackle a specific problem in a locally-relevant manner. For these reasons the King et al [20] definition of CE was introduced during the aforementioned interdisciplinary workshop in Nepal and we now adopt it as our formal definition of CE throughout this paper.

Community Engagement: *‘A participatory process through which equitable partnerships are developed with community stakeholders, who are enabled to identify, develop and implement community-led sustainable solutions using existing or available resources to issues that are of concern to them and to the wider global community.’*

This definition is important because, although CE appears in cross-disciplinary literature the extent to which the community is engaged in research can vary dramatically from filling in a questionnaire to co-producing an output, such as a policy brief or piece of art [11,18,19,21–24]. This methodological variability has led to CE being seen as interchangeable with terms such as ‘outreach’, ‘public engagement’, ‘awareness raising’, ‘participatory research’, even ‘education’ [11,16,25] which concerned our co-producers because they interpret the above terms, and their potential impacts, in specific and different ways. Whilst there is great value in outreach, awareness raising and other styles of intervention, from our perspective these are separate approaches to CE.

## Methods and results

The workshop sought to understand what co-producers considered as key *values* and *principles* underpinning community-based research, using an inductive thematic approach [26] to analyse this learning. An opening interactive session primed delegates by asking; ‘*what determines how you work when developing a CE project or* activity? *This could be resources such as money, time, and place, challenges to the working environment such as gender and intersectionality issues, but also ways of working such as interdisciplinary collaboration and co-production*’.

This question was unpacked through discussions and summarised into 7 areas; ‘*who we work with*’, ‘*institutions*’, ‘*scalability*’, ‘*creativity*’, ‘*power*’, ‘*evidence*’ ‘*interdisciplinary*.’ Groups of approximately six co-producers discussed how each area influenced their research, and what challenges and opportunities they posed. Six individuals volunteered to facilitate these discussions and remained on a single table whilst all others had the freedom to move between tables (but could remain at one if they so wished). After approximately 40 minutes of discussion facilitators fed back these reflections to the group. As such co-producers essentially began a thematic analysis because discussions were summarised and shared with the entire room inviting feedback and comments on this summary. Two note-takers recorded discussions and the whole event was filmed providing the data sources for the next stages of the analysis to determine the key *values* of community engagement research to tackle AMR (Table 1).

**Table 1.** The key values which support community engagement initiatives. Each value is underpinned by a series of principles (column 2) and suggestions and actions for achieving each are discussed (in column 3)

Value	Guiding principles	How do we achieve this?
Clarity	The project will deliver simple messages around a clearly defined problem.	All stakeholders will seek to understand our problem from as many different viewpoints as possible.
		The community will identify context-specific challenges facing them with regards to this problem.
		Involving all stakeholders from project design to dissemination will allow the development of concise messages relating to the problem.
		Final outputs and messages will be shared in a way that is ethically sound, simple and appropriate within our community.
	The project encompasses a defined community	All stakeholders will understand how we define our community and its boundaries.
		Researchers will explain the necessity of project partners and the inclusion of a wider stakeholder network.
	All stakeholders know what to expect from the project.	Researchers will be responsible for maintaining open lines of communication with all stakeholders throughout project planning, development, implementation and evaluation.
		All stakeholders will be involved in project design giving autonomy over each stakeholder’s level of involvement.
		Final outputs will be co-produced by the community, supported by other stakeholders to ensure for example, that accurate health information is being communicated.
Creativity	This project uses the most appropriate methods to formulate and communicate its key message(s).	The community will advise on what style of communication and learning are feasible, trusted and culturally appropriate for this project.
		The project will be engaging, meaningful and enjoyable for all stakeholders.
	The project is innovative.	Stakeholders will value resources that already exist in our community (people, groups, physical and digital materials, community motivations etc.)
		Community and contextual knowledge will shape project design, meaning we may utilise new or existing methodology, or a combination of both.
	Where creative outputs are produced the project places value on artistic form	All stakeholders will consider, and can explain, why a specific artistic form is appropriate to address this problem in this community.
		Stakeholders will work with personnel who have expertise in the chosen artistic form in order to co-produce final outputs.
		All stakeholders will showcase creative outputs as widely and freely as possible. For example; free exhibitions or via social media.
Evidence-led	The project addresses a recognised and defined problem	Stakeholders will have local, national or global evidence (or evidence gaps) to support the existence of our problem from multiple sources. E.g.: community dialogues, academic literature, grey literature.
		The community will have space to consider evidence and explain how the problem relates to their everyday lives.
	The project considers existing and previous approaches to solve this problem	Stakeholders will discuss what is currently being done to address this problem, what has (not) worked and in what contexts.
		Stakeholders will co-design an approach which considers current best practice, existing evidence and contextual factors specific to this community.
	The project is impact-led	Researchers will develop an evaluation programme alongside project development, not as an ‘add-on’ at the end.
		The evaluation programme will have multiple points of contact with each stakeholder, allowing data collection during the project, not just at the end.
		The evaluation programme will capture opportunities for improvement as well as evidence of success.
Equity	The project facilitates equitable partnerships with, and between all stakeholders	All stakeholders will recognise that equitable may not always mean equal due to existing power relations within the community, research team and stakeholder network.
		The insights and expertise of each stakeholder will be valued equitably allowing each to have as much autonomy as possible during project.
		Where creative outputs are produced, stakeholders will place equitable value on this artistic form as we do the health and behaviour-change outcomes.
	The needs, interests and values of the project are defined collaboratively by all stakeholders	All stakeholders will understand that the community are experts in their own lives, and respect their traditions, social norms, opinions, ideas and solutions
		Project development will be a collaborative effort with all stakeholders included in decision making processes, able to suggest changes and feel their contributions are valued.
		Academic and non-academic outputs and impacts will be valued, and invested in, equitably.
	Power-balances and how these may impact the project are considered and mitigated where possible.	Stakeholders will discuss the personal and professional relationships, cultural and social norms of our community, how these may affect the project, and implement solutions where possible to avoid barriers to engagement.
		Stakeholders will assess the political, cultural, social and geographical power landscape around our community and use this to inform equitable project design, implementation and dissemination.
Interdisciplinary	The project team, and management structure are configured based on the competencies required to address the problem.	All stakeholders will consider, utilise and respect the existing resources within the research team, community and wider stakeholder network.
	The project values non-academic partners.	All stakeholders will be open to expanding our network during project development, implementation and evaluation. For example; bringing in specialists in the development of a certain output.
	The project is guided by One Health approach	Stakeholders will consider if and how our methods and outputs will impact, or be impacted by, the Sustainable Development Goals (SDGs).
		Stakeholders will put our problem in to the context of the One Health Ecosystem surrounding it.
		Stakeholders will consider what risks can be mitigated, and outcomes maximised by involving additional disciplines.
	The project is evaluated by methods which allow its impact to speak to multiple disciplines	Researchers will employ a mixed-methods evaluation approach.
		Stakeholders will reduce discipline-specific jargon in all outputs (e.g. creative outputs, impact reports, conference presentations, academic publications, policy briefs)
		Stakeholders will ensure our findings are shared across the community in ways that are meaningful to them. I.e. at town council meetings.
Sustainability	This project (or parts of it) can be scaled-up or expanded to reach a wider audience.	When reporting on the project researchers will consider how it can be applied to different geographical/social/cultural and One Health contexts.
		Stakeholders will share our methodology, best practice and challenges as widely as possible within *their* networks. For example; a project manual in local languages can be shared beyond our initial community.
		Researchers will publish in open access journals to allow the project to reach as wider academic audience as possible.
	The project (or parts of it) can be sustained long-term.	Where possible project design will facilitate a community legacy regardless of continuing funding/support. This ensures outputs and/or impacts of the project can reach a wider audience.
		Where possible stakeholders will consider the financial and capacity needs to support a community legacy or maintain this project in the long-term.
		By maintaining connections with our community and wider stakeholders, researchers can place themselves in a strong position to monitor long-term impacts of the project.
Flexibility	The project is an iterative process and responsive to the needs of all stakeholders.	Researchers will understand that the needs of the community in relation to the problem may change during the project and have capacity to react to these changes.
		All stakeholders will understand the financial, temporal and topical limitations of this project and respect this when suggesting changes.
		All stakeholders will understand that they can question and drive changes within the project.
	The project is a learning experience for everyone involved.	New learnings which could lead to different outputs will be discussed thoroughly before the project is modified.
		All stakeholders will be aware that additional partners may need to be recruited during the project.
		The evaluation programme will enable active learning by capturing stakeholder views during the project not just at the end.
	The research team value time as a key resource to maintain flexibility.	Researchers will ensure the project timescale allows for regular reflection from all partners.
		All stakeholders will facilitate an open culture for the discussion of project development.
		All stakeholders will assess and communicate risks as they appear, recognising that this may halt or slow down project development.

**Table 2.** Exploring the values and principles in action through four case studies of current research utilising community engagement within AMR

CASE STUDY PROJECT DETAILS	PROJECT SUMMARY	STRENGTHS HIGHLIGHTED BY TOOL	CHALLENGES HIGHLIGHTED BY TOOL
1. SOURCING COMMUNITY SOLUTIONS TO ANTIMICROBIAL RESISTANCE, NEPAL. UNIVERSITY OF LEEDS AND HERD INTERNATIONAL (2016–2019)	This project (28) utilised participatory film-making to explore community-level relationships with antimicrobials across two sites in the Kathmandu Valley of Nepal. Stakeholders included filmmakers, pharmacists, anthropologists, and health professionals contributing to an interdisciplinary project with creativity at its heart. Participants took part in a week-long workshop to discuss their understanding of antimicrobial medicines and how these were used within their community. Focus then shifted to the concept of AMR, and what behavioural actions the community took which could be driving resistance (e.g. failing to finish a course of antibiotics, drinking milk from cows undergoing antimicrobial treatment etc.). Running in tandem with these information-based sessions were workshops supporting participants to develop their own narratives around antimicrobial use and AMR. Participants were trained to make short films, which were shared at community showcasing events and, since the completion of the project, have been shown to local government and Ministry of Health and Population officials.	**Evidence led**: The team invested in a pre-testing phase where stakeholders considered the community language around antibiotics and to introduce the scientific terminology to be used in the final iteration of the project. This allowed all stakeholders to appreciate each other’s level of understanding around antimicrobials and AMR, and to ensure the final intervention was specifically tailored to each community which required flexibility in language and delivery style. **Creativity**: This projects’ emphasis on learning filming techniques early on took the focus away from the ‘teaching’ of AMR facts. Instead participants absorbed this information whilst reflecting upon their own experiences and developing their stories, characters, and films. The resulting outputs are engaging short films which have value as an artistic form in their own right, not just as health advocacy tools. **Sustainability**: The project has had a degree of policy-level impact. Local government officials have recommended the films be shared within schools (a follow-on project has recently been funded to do so), whilst the Ministry of Health and Population has asked HERD International to support the development of Nepal’s national AMR action plan. The tool’s emphasis on sustainability allows this impact to be captured and realised in a way that quantitative evaluations may not.	**Equity**: The tool highlighted the challenges this project faced around giving participants freedom to develop their antimicrobial stories and films. Although autonomy for participants was crucial, inaccurate health-related information had to be corrected by the research team to avoid misleading the films’ audiences. The project team took a strict approach to their internal training, ensuring all facilitators had a clear understanding of AMR. Linking to the value of clarity, an AMR fact sheet was provided for participant-facing work and corrections of misinformation were made immediately as the situation arose. This ensured participants received clear health-related information consistently, and that any corrections were made in an understanding way so as not to risk the disengagement of participants.
2. DUST BUNNY: UNDERSTANDING THE HOME AS A SOURCE OF INFECTION OF AMR BACTERIA CARRIED BY DUST IN GHANA. LANCASTER UNIVERSITY AND NOGUCHI MEMORIAL INSTITUTE FOR MEDICAL RESEARCH (2018 – PRESENT)	Dust Bunny provides an informed assessment of societal practices in domestic cleanliness and hygiene and co-creates locally appropriate solutions aimed at reducing infections in the home. The project explores hygiene practices across different home environments, to understand bacterial communities, and the extent to which AMR is driven by household practices (32).	**Evidence-led**: Some localities had been exposed to many research teams leading to criminals gaining entry to people’s homes, under the guise of a research project, and stealing valuables. Dust Bunny took on board this evidence and tackled the problem by providing the Ghanaian Researcher institution phone numbers (and mobile phones) so that homeowners could directly call the university before letting a researcher into their homes. This approach really exemplifies the value of being evidence-led and also allowed participants a sense of equity within the research process as they could directly communicate with the UK and Ghana-based teams. **Creativity**: Dust Bunny was challenged on how to remunerate participants. The team was keen to place value on people’s time commitment but was worried about offering financial rewards which could place community members vulnerable to theft. The team took a creative approach by offering a batch of inexpensive but useful cleaning products as remuneration. This really embodies the value of creativity but also clarity as the reward of cleaning products clearly links back to the project main aims of enhancing home hygiene.	**Clarity**: Dust Bunny works as a consortium of stakeholders from very different backgrounds (microbiology, design, home hygiene, public health). As such clear communication around the medical terminology used in this project was essential to ensure all stakeholder could communicate. The team reflect that they were challenged in terms of clarifying their language from the initial proposal phase right through to the community engagement interventions where lay language around antimicrobials had to be agreed up on. Some project outputs may still utilise differing terminology now depending on the intended audiences. **Equity**: in Ghana, females predominantly clean the home. Hence, to fit with social norms the community-facing team were recruited to be female-biased. However, the research team was male-biased and this experience of managing gender at the community level has caused project leaders to reflect upon their recruitment and training opportunities at their home institutions. Had this tool been available at the start of the project, the team reflect gender-balanced recruitment in the research team could have been more equitable.
3. SUPPORTING EVIDENCE-BASED POLICY: A LONGITUDINAL STUDY OF AMR RISK BEHAVIOURS AMONG LIVESTOCK KEEPING COMMUNITIES IN INDIA AND KENYA. ROYAL VETERINARY COLLEGE. (2017–2019)	This project engaged livestock-keeping households in Indian subsistence dairy farming communities and rural Kenyan pastoralist communities. Both were previously involved in research on animal health which yielded long-term datasets on AMR-related behaviour and which the project team intended to use as a tool to forecast AMR. Researchers also engaged with children in these areas through the production of comics to tell locally-relevant AMR stories, raising awareness of AMR and the principles of good antimicrobial stewardship. This aspect of the project became a much larger component than originally intended and the project team’s commentary on *Values* and principles of CE specifically refer to this creative aspect.	**Creativity**: The team was proud of the emphasis it put on the ways in which the project engaged culturally appropriate artistic forms. Comics in India are a popular, well-used medium for communicating with young people. In Kenya they remain novel, especially in rural settings, which makes them particularly exciting to young children. As such, comics were considered appropriate for communicating challenging messages to 10–14-year-old children, if for slightly different reasons in each setting. **Interdisciplinary**: the team engaged with local illustrators to design each comic and brought the children into this feedback loop, in so doing creating an interdisciplinary experience including non-academic knowledge. All designs, characters, plots, and activities were assessed by children in focus group discussions allowing the creative process to be owned by all research participants. There were also interdisciplinary learning opportunities. For example, illustrators did not have specific backgrounds in creating educational material, so the project was a chance to develop these creative processes.	**Clarity**: This project was specifically challenged by the second *principle* underlying the *value* of clarity: ‘*The project encompasses a defined community’*. 10–14-year-old children were initial defined as the key community. However, as the comics became so popular their dissemination was extended to communities beyond the initial age-range and locality, meaning the stories and characters may not have been as contextually appropriate to these new audiences. This was particularly so for the Kenyan comics, which were later shared in urban settings as part of National Antimicrobial Awareness Week 2019. Here they reached an audience of mixed ages who may not have been so closely connected to agricultural situations. **Sustainability and flexibility**: In reference to the example above, the tool also highlights how this project was challenged to meet the values of sustainability and flexibility. The project team reflects that had they considered sustainability as a key value at the project development stage it may have prompted earlier discussions on the potential scale of comic dissemination. This could then have prompted some flexibility in how to manage the specificity of their content.
4. LIFTING THE LID ON BACTERIA’: DESIGNING AMBIENT COMMUNICATIONS TO IMPROVE HAND HYGIENE IN PRIMARY SCHOOL TOILETS. UNIVERSITY OF LEEDS (2017–2019)	This design project investigated the potential of ambient communications in primary school toilets to improve hand hygiene practices. Ambient communication involves the unexpected integration of graphic messages in specific environments. Usually employed by commercial companies to improve engagement with a product, this study had ambient communications co-designed by primary school children for use in their toilet facilities.	**Flexibility**: At the start of the project the research team shared existing handwashing communications with children to understand what visuals and messages they liked but checked-up on this throughout the project in focus groups. Hence, researchers primed children with knowledge from existing evidence but were also themselves primed by the motivations and experiences of the children. This co-priming balance is considered the basis for flexible, equitable and evidence-led co-design projects. **Interdisciplinarity**: The tool highlighted both the strength and challenge of interdisciplinarity for this project. A mixed methods evaluation considered both qualitative interview data, and quantitative measures of soap usage and bacterial hand swabbing. This mixed data has been beneficial in terms of the projects’ appeal to interdisciplinary audiences. For example, health professionals can realise the benefits of the design tools via the 60% increase in use of hand soap (21, 24). Yet design professionals can reflect upon the importance of the participatory approach to creating the graphics with children.	**Interdisciplinarity**: Despite the strengths of their mixed methods evaluation (Strengths column), this team worried that reviewers from discrete disciplines, such as health or design, may focus on certain aspects of the study rather than taking a holistic view of the interdisciplinary methods and evaluation used. They reflect that deciding on journals for publication was time-consuming and this represents a challenge for the *value* of interdisciplinarity that is likely common across CE projects. **Equity**: Allowing children to have creative freedom in their designs fostered ownership. However, the design team often noticed promising ideas and had to find creative and equitable ways of developing these without alienating other children or forcing the direction of design. The role of wider stakeholders was key to this process. For example, teachers identified that some of the children’s designs were inappropriate for use in the school toilets and advisory board members could stress the importance of all stages of hand hygiene including drying, which was missed in some designs. The research team related to the value of equity through reflecting on these moments of tension.

Following the workshop, a single researcher first analysed the key themes of discussions, from notes and films. Themes were clarified and adapted as mind maps and table voice recorder data were analysed. The final round of analysis considered recordings and notes of discussion sessions from throughout the workshop.

From this analysis, seven *values* emerged, each underpinned by a set of *principles. Principles* appear as sub-themes, based on co-producers’ discussions of ways of working which would facilitate the overall *value* being incorporated into AMR interventions (Table 1). The process then became iterative. A draft of Table 1 was sent to co-producers for feedback, amended and re-circulated, allowing co-producers to revisit and reflect [27] upon the *values* and *principles* within the scope of their own projects. Key amendments to the initial language included removing the word ‘empowering’ which was seen by many co-producers as a top-down and patronising way to view one’s community. Academic jargon was removed to facilitate translation and thus allow all stakeholders to engage with the tool regardless of background. Following this stage of reflection, four projects (working in different contexts) were asked to produce short case studies to exemplify the *values* and *principles* in action.

### Defining the values and principles of successful community engagement projects

**Clarity**: Throughout discussions, plenary sessions and presentations, the issue of clarity was of paramount concern. From a research perspective, co-producers stressed the importance of focusing on clear questions and communicating these openly with other stakeholders to avoid over- or false-promising on outcomes. From the practitioner perspective, there was a focus on the use of simple, locally-relevant language to communicate with wider stakeholders for which AMR may be a novel term. Finally, in the community, the onus was on clarifying the needs and expectations of the project based upon lived experiences and the local context of the AMR challenge. Linking to the *value* of flexibility, co-producers felt it was important to create space within the project timeline for discussions. This ensures knowledge can be shared between stakeholders whilst keeping focus on the project’s aims and everyone’s roles within it.

**Creativity**: Co-producers were keen for project design to be question-focused and to utilise methodologies that are familiar to stakeholders. However, there was also acceptance that certain methods may be better suited to answering certain questions and so this *value* does overlap with the next (being evidence-led). There was agreement on the huge benefit to having artistic practitioners (filmmakers, theatre producers and graphic designers) in our co-production team with respect to this *value*. The group stressed the need to ensure that, where creative outputs are developed, value is placed on the artistic form being used. This extended from the project-planning phase where discussion should focus on *why* a specific form (drama, film etc.) is appropriate, through to dissemination. For example, the film outputs of Case Study 1 [28] have integral creative and social value as well as being effective AMR resources or tools.

**Evidence-led**: Understanding previous, current and emerging work on AMR was a common theme of co-producer discussions. Many stressed the need to look beyond academic publications for evidence, and to value the expertise and lived experience of one’s community who are experts in their own lives, particularly their health seeking and hygiene behaviours which likely influence AMR. Preliminary dialogue with the community and wider stakeholder network was seen as crucial to formulating AMR research questions and deciding on methodology, as exemplified by Case Study 4 [29–31]. Pilot and pre-testing phases were encouraged to ensure methods and approaches best fit the context in which one is working. Finally, co-producers discussed the need to consider the evidence produced and how this can best be shared and made accessible after the project is completed. Evaluative methods prompted lengthy discussions with randomised control trials seen as important in providing quantitative evidence for success, but criticized for not taking into account the complexities of AMR and the often potentially far-reaching impacts of CE beyond the defined outcome of the trial. A general consensus was that mixed-method approaches can balance quantitative and qualitative evidence. This not only provides a more robust assessment of the impact of a CE intervention, but also allows findings to be appreciated by interdisciplinary audiences and widens the reach of the intervention.

**Equity**: Co-producers overwhelmingly stated that within CE the voices of all stakeholders should be appreciated, but this may not always be equally weighted hence the *value* of *equity*. Although researchers will be seeking to co-develop AMR solutions with their community partners, clear and accurate health-based information on AMR must be provided and inaccurate comments corrected by the research team. This must be handled fairly so as to balance power dynamics between stakeholders. Many co-producers stated that involving the community in project planning was a successful way of ensuring equitable partnerships developed from the offset as the community, research team and wider stakeholders could share existing knowledge and have their assumptions challenged within a safe space. Consideration of social norms in the community is also important with regards to equity, for example, Case Study 1 [28] realised through focus groups that there were gender differences in health-seeking behaviours which may impact on AMR. These findings shaped not only the way this project was evaluated but also allowed a follow-on project to consider gender differences in more detail. Finally, co-producers discussed equity challenges with how research councils initially award funds to the Global North partner, and require Global South partners to complete a lengthy due diligence process. This can lead to unequal expectations that power ultimately lies with Global North institutions. For Case Study 2 [32] this caused challenges with Global South partners feeling less confident to take the lead on project development. However, regular meetings, where Global South expertise was explicitly and demonstrably valued worked to lessen this expectation over time.

**Interdisciplinarity**: AMR impacts on human, animal and environmental health. Thus, it is almost impossible to address via a single discipline. Case Study 3 considered the use of antimicrobials in rural Kenyan farming communities but, because humans and animals share the same water sources in these locations, it was difficult to attribute AMR at sampling sites exclusively to livestock. Through creative engagement with people (comic book development and interviews) the team began to understand the behaviours which underpinned their biological data. Co-producers also stressed that community knowledge represented interdisciplinarity. For example, is it appropriate to advocate for prescription-only antibiotic use in a community that lives several days walk from a medical centre? Community stakeholders are best-placed to answer this question. Academic interdisciplinarity was seen as actively encouraged by research councils via their development of cross-cutting funding calls. Understanding the experience of different team members in terms of the value it brings to the project’s research questions, rather than hierarchy or standing within a given professional research community, was considered a key approach to facilitate project-wide interdisciplinarity. Considering the Sustainable Development Goals could also drive interdisciplinary focus, particularly in terms of troubleshooting problems arising from presumed solutions. Finally, linking to the *value* of being *evidence-led*, co-producers stressed the need to evaluate projects in a way that was relevant to multiple disciplines so that learnings can be shared more widely.

**Sustainability**: A key challenge for co-producers was the ethics around what happens to a project when the funding ends. One Health issues are rarely solved by a silver bullet and CE, in particular, is invariably a slow-burning solution realised through incremental changes in behaviour. Co-producers discussed the need to ensure communities have strong ownership of a project, allowing them to visualise how resources and skills could be used beyond the funding lifespan. The development of equitable partnerships built upon joint interests also appeared in the discussion of this *value*, as did utilising existing stakeholder networks to ensure that solutions are taken up and embedded in the longer-term strategy of community-based organisations. As researchers, it was felt that this approach to CE projects sowed the seed for continued relationships beyond the funded phase of the project and facilitated long term evaluations of the work. This *value* resonates with that of equity and being *evidence-led*, since for a community to take ownership of a project and its outputs, they must be fully invested in the project and not view it as a tokenistic opportunity.

**Flexibility**: Flexibility was deemed essential to managing expectations and ensuring positive outcomes for all stakeholders. AMR is driven by multiple dynamic factors, many of which are poorly understood, including the environmental burden of AMR. As such research questions relating to AMR have the potential to change mid-project with no regard for planned outcomes and impacts. CE is a flexible approach which is iterative in nature, allowing stakeholders to modify methods and outputs as they learn throughout the project. However, communication of this flexibility was seen as paramount from the researchers’ side. A community may be looking to researchers or other stakeholders for clear guidance and defined answers. Challenging this expectation early on in the project was seen as important to allowing flexibility to be accepted across stakeholders facilitating the iterative development of the project itself and supporting the *values* of clarity and equity.

### Tool overview

To bring these *values* to life and actively assist the design, development and evaluation of successful CE projects, we have created a practical tool. Table 1 shows each value, their underlying *principles* and additional guidance on how to achieve them. This tool is intended to be used by all stakeholders as a roadmap to ensure each CE project is upholding the seven *values* of Clarity, Creativity, being Evidence-led, Equity, Interdisciplinarity, Sustainability and Flexibility.

## Case studies

To consider the *values* and principles of community engagement in action we discuss four case studies of CE for AMR projects, dissecting how each *value* (or *principle*) is achieved (Table 2). Case studies are of existing or recently completed projects developed by the same network of researchers and practitioners who co-produced the *values and principles* tool. For the full case studies see the supplementary material.

## Discussion

### The value and challenge of applying community engagement approaches to AMR

The tool presented in Table 1 is intended to support research which addresses One Health challenges, such as AMR, by fully engaging and working with the community the research is intended to benefit. This tool provides a set of key *values* to direct community-engaged AMR research and underpins these with *principles*, which act as indicators, allowing teams to track which *values* they are achieving, and the comparable level of coverage of each *value*. We foresee the tool being utilised to support existing frameworks [9,23] of CE or participatory research including Arnstein’s ladder of participation [13] but with a specific focus on AMR and One Health challenges. A limitation of the tool is that it was created during a single workshop and utilising only this network of approximately 40 co-producers. That said, the network was diverse and included CE experts from a range of disciplines who were focusing on health challenges including AMR, Mental Health, neglected tropical diseases, and maternal health. As a result, although the values, and most *principles*, are applicable to CE projects in general, some consider the specifics of the AMR challenge. For example, a *principle* underpinning the *value* of interdisciplinarity: ‘*The project is guided by a One Health approach’* points specifically to the complex dynamics of AMR which include human, animal and environmental health all of which are inextricably linked. As such, this tool is focused toward a One Health application of CE methodology and will need adapting to alternative settings, for example, education.

The case studies summarised in Table 2 demonstrate how existing CE projects, led by co-producers of this tool, achieve, and are challenged by the *values* at various stages. However, the tool is not intended to simply evaluate work which has already occurred. Rather, we aim to present a flexible framework for applying CE projects to the field of AMR and One Health at all stages of the research process. It is hoped that by presenting this reflection of existing work, alongside the tool itself, users can appreciate its flexibility, and foresee troubleshooting opportunities rather than using the tool as a tick-box exercise. Indeed, co-producers agreed that few CE for AMR projects will naturally meet all *values* and *principles*. There will be particular challenges in terms of balancing participants’ creative freedom with project aims, of being flexible within funding constraints, and in creating an output that is both artistically valuable and scientifically/medically accurate. However, using this tool as a guide to shape project development should allow all stakeholders to foresee potential challenges and work toward solutions.

An additional point of agreement was the implicit assumption of trust. All co-producers were unanimous in considering trust between and within stakeholders as integral to successful CE. However, trust cannot be instantly given or simply ticked off a list. It must be developed over time. These discussions support findings of the initial workshop in Nepal where trust did not appear as a key *value*. According to co-producers this exemplifies the need for trust to be developed organically and fed by the collaborative nature of the CE method. If these *values* are used from project planning onwards, trust can develop intuitively, and space will be created to allow all stakeholders to feel both trusted and trusting.

Workshop notes and transcripts helped fine-tune the tool as they revealed conversations on why CE is appropriate to AMR, but where the challenges lie in practically implementing this approach. CE is valued in One Health because of it’s potential to bridge gaps between research and practice [21] and to ensure power is held at community level [22,24]. However, methodological support for designing and implementing CE methodology is sparse [33–35]. Co-producers considered this broadly problematic because it means best practice and troubleshooting guidance are not shared. This could lead to the formation of weak CE approaches where the community may not be truly engaged, or benefit from their involvement [23,36]. There can be unintended negative consequences of CE interventions [37–39] which could be particularly harmful in the context of AMR. An example discussed by co-producers, and linking to the value of Clarity, was that if AMR – itself a complex issue – is not communicated in appropriate local language, communities may misunderstand and falsely believe that all antimicrobial use contributes to AMR. This may cause reluctance to take medication, putting communities at risk of easily preventable diseases. Co-producers worried that a lack of methodological guidance, combined with serious repercussions of unintended negative consequences, could detract new teams (such as those working on AMR) from attempting CE. Concerns were frequently discussed in relation to cost. CE projects can be resource heavy in both time and money, thus adding in risk factors described above can make them difficult to justify. During the workshop such conversations confirmed the need to develop a tool (Table 1) rather than a briefing paper alone, as co-producers were unanimous that practical support was needed to align AMR challenges with the CE approach.

Because AMR is often a locally specific and complex problem, co-producers strongly agreed that CE has the potential to build contextually appropriate solutions. This is reflected within the *values* of Equity, Clarity and (being) Evidence-Led, and wider literature which considers CE to value local knowledge, foster a sense of trust between stakeholders, and build specific solutions which hinge on changing behaviours at very local levels [16,18,33]. However, specificity was considered a double-edged sword because CE projects tend to become so focussed on their context that it can be difficult to realise common synergies across projects. There is currently limited collective discussion of data collection, analysis and measure of success which means comparing the impact of projects is problematic [36]. Co-producers suggested this lack of evaluative support could curb the enthusiasm of researchers to utilise CE methods, of awarding bodies to fund CE approaches, or for policy (and other decision) makers to trust the findings of such projects [22]. Additionally, AMR projects often take a holistic approach to evaluation such as Case Study 4, which measured health/hygiene outcomes and evaluated the confidence of participants. Such projects may be difficult to place and search (in the literature) if not labelled correctly as their methods of data collection, analysis and evaluation are either field-specific or (too) highly interdisciplinary [33,35]. It is hoped the *values* and *principles* presented here will allow projects to capitalise on the potential of CE to tackle AMR challenges, and support them to consider context and disciplinary reach when deciding on evaluative methods. In combination this should allow robustly designed and evaluated projects which best serve their community’s needs, but also evidence the scope of CE to address AMR.

### Concluding remarks

This *values* and *principles* tool has been co-produced by over 40 researchers and practitioners who utilise community engagement approaches to tackle One Health issues. It was created in response to both the growing threat of AMR, and the growing realisation of AMR as a social issue which can, at least in part, be addressed by community-based interventions. Unfortunately, as discussed by co-producers, language barriers and limited methodological support for CE means it is currently under-utilised within the AMR sphere. To bridge this gap, we propose this tool be used to guide the development of clear, creative, evidence-led, interdisciplinary, equitable, sustainable and flexible research which can support multiple stakeholders to tackle an AMR problem collaboratively and through a locally meaningful intervention. However, this tool is not prescriptive and the methods by which a team decides to approach each *principle* and *value* are entirely plastic and should be driven by local context. Our co-producers have exemplified this by providing Case Studies of the tool in action, in so doing reflecting upon their current CE for AMR projects. Case studies demonstrate the different ways each *value* and *principle* can be achieved but also the key challenges a project team can face. CE is popular and appropriate within the broader sphere of Health interventions and has the potential to revolutionise AMR research. However, it is no silver bullet and it cannot be used formulaically. All stakeholders must respond to the local context around their problem and be prepared to listen, learn, and reflect throughout the process. It is hoped this tool can encourage and provide practical support for high quality community engagement interventions which positively impact the complex challenge of AMR.

## Paper context

Community engagement (CE) approaches are used to meaningfully engage people and create positive behaviour change. However, as yet these methods have been under-utilised in the field of AMR. We summarise discussions with a group of experts on the potential for CE to be utilised within AMR. We present key values and principles underpinning CE in a tool designed to guide interventions in the AMR sphere, and exemplify this with recent case studies.
